# Time-of-flight and noise-correlation-inspired algorithms for full-field shear-wave elastography using digital holography

**DOI:** 10.1117/1.JBO.26.8.086006

**Published:** 2021-08-19

**Authors:** Agathe Marmin, Gabrielle Laloy-Borgna, Sybille Facca, Sylvain Gioux, Stefan Catheline, Amir Nahas

**Affiliations:** a The University of Strasbourg, ICUBE Research Institute, Strasbourg, France; b LabTAU, Inserm U1032, Lyon, France; c University Hospital of Strasbourg, FMTS, ICube CNRS 7357, University of Strasbourg, Department of Hand Surgery, SOS hand, Strasbourg, France

**Keywords:** elastography, transient elastography, holography, quantitative, shear-wave, noise-correlation

## Abstract

**Significance:** Quantitative stiffness information can be a powerful aid for tumor or fibrosis diagnosis. Currently, very promising elastography approaches developed for non-contact biomedical imaging are based on transient shear-waves imaging. Transient elastography offers quantitative stiffness information by tracking the propagation of a wave front. The most common method used to compute stiffness from the acquired propagation movie is based on shear-wave time-of-flight calculations.

**Aim:** We introduce an approach to transient shear-wave elastography with spatially coherent sources, able to yield full-field quantitative stiffness maps with reduced artifacts compared to typical artifacts observed in time-of-flight.

**Approach:** A noise-correlation algorithm developed for passive elastography is adapted to spatially coherent narrow or any band sources. This noise-correlation-inspired (NCi) method is employed in parallel with a classic time-of-flight approach. Testing is done on simulation images, experimental validation is conducted with a digital holography setup on controlled homogeneous samples, and full-field quantitative stiffness maps are presented for heterogeneous samples and *ex-vivo* biological tissues.

**Results:** The NCi approach is first validated on simulations images. Stiffness images processed by the NCi approach on simulated inclusions display significantly less artifacts than with a time-of-flight reconstruction. The adaptability of the NCi algorithm to narrow or any band shear-wave sources was tested successfully. Experimental testing on homogeneous samples demonstrates similar values for both the time-of-flight and the NCi approach. Soft inclusions in agarose sample could be resolved using the NCi method and feasibility on *ex-vivo* biological tissues is presented.

**Conclusions:** The presented NCi approach was successful in computing quantitative full-field stiffness maps with narrow and broadband source signals on simulation and experimental images from a digital holography setup. Results in heterogeneous media show that the NCi approach could provide stiffness maps with less artifacts than with time-of-flight, demonstrating that a NCi algorithm is a promising approach for shear-wave transient elastography with spatially coherent sources.

## 1 Introduction

The structure of biological tissues can be altered by certain pathologies, such as malignant tumors, metastasis, or inflammatory diseases. This change of structure leads to the modification of the mechanical properties of tissues whether it is local, in the case of a tumor,^1^^,^^2^ or affects the whole organ, for example in liver fibrosis.^3^ When the tissues are accessible, experienced physicians can detect those structural anomalies through palpation. The information provided by touch is thus imprecise and qualitative. Adding quantitative stiffness to medical imaging systems could be a strong aid in diagnosis, particularly in cases of small lesions or inaccessible tissues.

In the last three decades, elastography methods have been developed using ultrasound or magnetic resonance imaging in order to meet the need for an elastography imaging system for diagnosis.^4^^–^^6^ In the late 1990s, optical systems were adapted to perform elastography, allowing high-resolution and contactless surface measurements. Since Schmitt introduced elastography in optical coherence tomography (OCT) in 1998,^7^ different optical approaches to elastography have been studied.^8^^,^^9^ They can be classified as quasi-static,^7^^,^^10^^–^^13^ harmonic,^14^^,^^15^ or transient^16^^–^^29^ depending on the external mechanical stimulation used on the sample. The focus of this study is transient elastography.

By imaging the shear-wave propagation of a pulsed stimulation, transient shear-wave elastography provides a quantitative real-time measurement of the mechanical properties of the tissue, from the speed of the shear waves based on the imaging of displacements on the sample of interest.^14^ OCT-based transient elastography has shown impressive results on biological samples,^15^^–^^22^ notably in non-contact cornea imaging using air puffs.^23^^–^^25^ Full-field transient elastography has also been demonstrated on optical images,^26^ showing the feasibility of shear-wave propagation tracking in real time. To our knowledge, this makes transient elastography a very promising approach for clinical transfer.^27^^,^^30^

In the case of transient elastography, the data from the propagation movie used to be processed using an inverse problem algorithm.^28^^,^^29^ Currently, the most common processing methods used for performing transient elastography with optical systems are shear-wave time-of-flight-based approaches.^14^^–^^26^ In this paper, classic time-of-flight was used as well as a noise-correlation-inspired (NCi) method. This alternative method is based on recent work in passive elastography^31^^–^^34^ where a noise-correlation algorithm is used to process shear-wave speed from diffuse fields. We propose in this paper an adaptation of this noise-correlation approach for transient elastography with spatially coherent shear-wave sources. This NCi approach allows to perform full-field elastography without the need to specifically compute the propagation direction of the mechanical pulse, and can be used with any pulse temporal shape such as half-sine, chirps, or noise pulses. The NCi approach is also inherently robust to reflections. The time-of-flight and the NCi algorithms were tested on simulation images. Both methods were then combined with an off-axis digital holography setup, thus achieving high-sensitivity full-field phase and amplitude images from each frame. The proof of concept of the NCi method was performed using agarose samples mimicking biological tissues, and first results on an *ex-vivo* biological sample are presented along with classic transient elastography measures.

## 2 Materials and Methods

### 2.1 Shear-Wave Propagation in Biological Soft Tissues

When considering an elastic behavior in a soft medium, the mechanical properties of the medium can be characterized by the shear modulus. The shear modulus, denoted as μ, is defined as the ratio between the shear stress applied on the medium and the shear strain. Biological soft tissues are considered incompressible (Poisson ratio μ≈0,5). Consequently, the shear modulus can be linked to Young’s modulus, denoted as E, which is often used to characterize stiffness for biomedical applications:

E=3 μ. (1)

The mechanical waves propagating through a homogeneous infinite medium can be classified as compression waves or shear waves. For soft biological tissues, the speed of compression waves is around 1500  m/s with a small variability between the different types of soft tissues, whereas shear waves propagate with velocities ranging from 0.5 to 10  m/s.^8^ With these considerations, we examine shear waves’ propagation in this study. The phase velocity $c_{\text{shear-wave}}$ of shear waves propagating through a homogeneous, isotropic, and infinite media can be linked to the medium shear modulus μ and its mass density ρ:^35^

$$c_{\text{shear-wave}}=\sqrt{\frac{\mu }{\rho }}.$$ (2)

The density of soft biological tissue can be approximated at $1\;g/{\mathrm{cm}}^{3}$. Because the optical setup in this study images the phase and amplitude at the surface of the sample, the mechanical waves at the surface are Rayleigh waves. Considering a linear elastic medium, their phase velocity $c_{\text{Rayleigh}}$ is proportional to the velocity of the shear waves:^34^

$$c_{\text{Rayleigh}}≃0.95\sqrt{\frac{\mu }{\rho }}.$$ (3)

This relation demonstrates the possibility of retrieving the quantitative stiffness of soft biological tissues from shear-wave imaging assuming semi-infinite homogeneous media. If there is a stiffness change within the medium, the relation between the shear-wave speed and the medium stiffness could be modified: Eq. (3) might not be valid in the case of heterogeneous media.

### 2.2 Imaging Shear-Wave Propagation Using Digital Holography

**Fig. 1:**
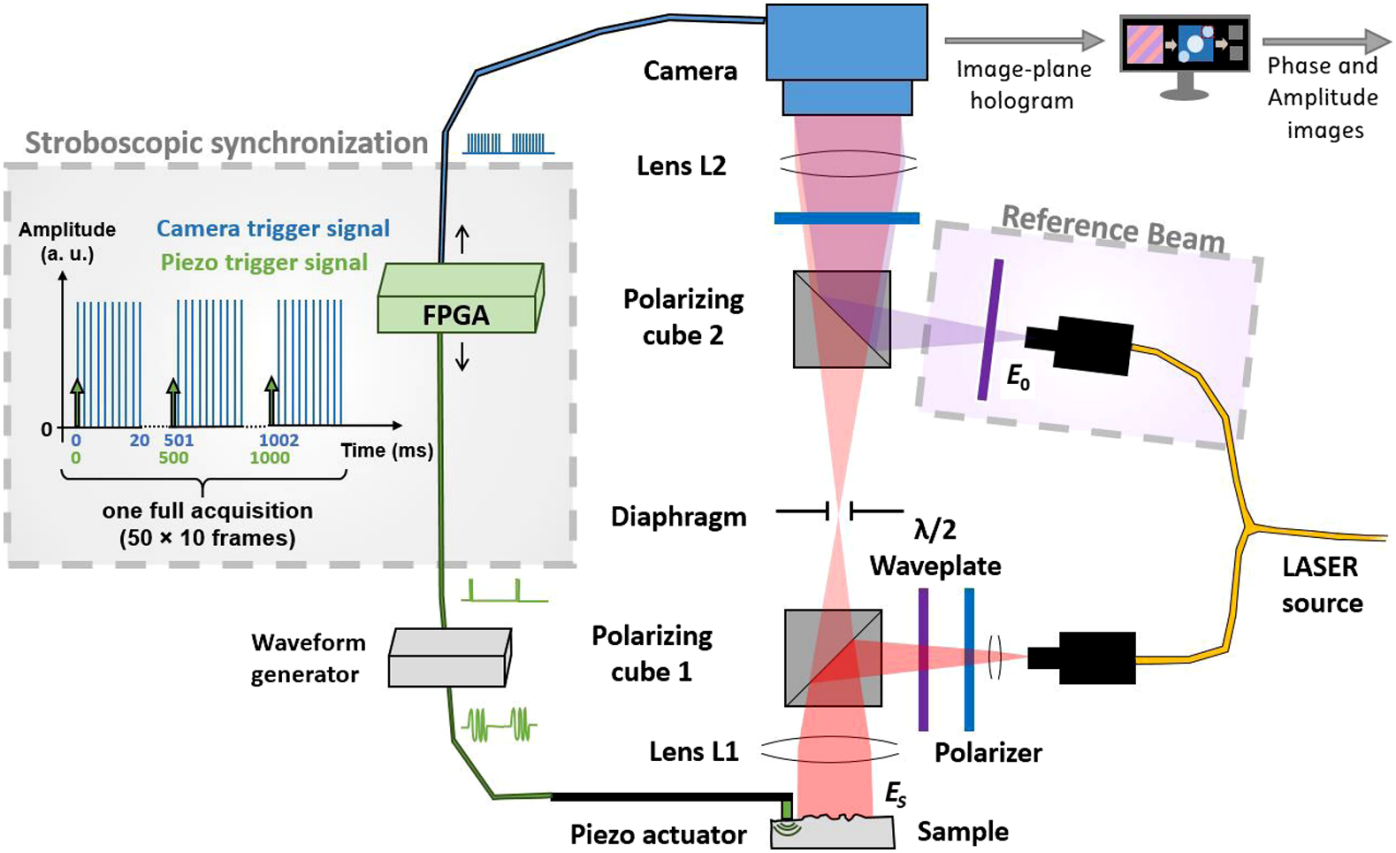
Schematic of the holographic off-axis setup and the stroboscopic approach. The optical setup is represented on the right of the image: the object beam (in red on the image) is back-scattered by the sample before interfering with the reference beam (in purple) ($f_{L1}=100\;\mathrm{mm}$, $f_{L2}=150\;\mathrm{mm}$). On the left of the image, the synchronization of the camera and the mechanical actuator is detailed: the impulses represented in green trigger the piezoelectric actuator and the blue signal activates the camera.

The setup in this paper is an off-axis interferometer based on a Mach–Zehnder, illustrated in Fig. 1.^34^ The light source is a 671-nm continuous single-mode laser (CNI MSL-FN-671-S) with a total power output of 250 mW and an $8\;\mathrm{mW}/cm^{2}$ power density on the sample. A 1440×1440  pixels complementary metal oxide semiconductor camera (Adimec, Q-2HFW) records image-plane holograms at 500  frames/s over a $11\times 11\;mm^{2}$ field of view. Using a two 2D Fourier transform method, the amplitude and phase images are extracted from each frame.^36^ The processed images of the surface of the sample have a lateral resolution of 50  μm and a 10-nm displacement sensitivity.

The propagation of surface waves in soft tissue being in the order of 1 m a second, and a stroboscope approach was used to reach an equivalent 25 kHz frame rate to image propagation over the field of view. We achieved this frequency by synchronizing our mechanical source with our camera using a field programmable gate array. Each acquisition consists of 500 frames, which are acquired 10 at a time. Between each 10-images acquisition, the time delay between the generation of the mechanical impulse and the image acquisition is slightly extended. This difference corresponds to the period of the equivalent frame rate, here 25 kHz.

A synchronized piezoelectric actuator (Thorlabs, AE0505D08F) was used to transmit mechanical impulses of hundreds of nanometers in amplitude to the sample. The impulses were transmitted to the sample through a metallic plate with a $10\times 1\;mm^{2}$ section in contact with its surface. The generated vertical displacement at the surface of the sample is denoted as $\psi_{z}$. The phase ϕ retrieved by the holography setup can be expressed as

$$\phi (x,y,z)=\frac{4\pi n\psi_{z}}{\lambda_{\text{Laser}}}+\phi_{\text{noise}}(x,y,z)+\phi_{\text{speckle}}(x,y,z)[2\pi ],$$ (4)

with n the refractive index of the medium at the interface with the sample, $\lambda_{\text{Laser}}$ denotes the illumination wavelength, $\phi_{\text{speckle}}$ denotes the speckle noise, and $\phi_{\text{noise}}$ denotes other noises, notably from the light source and the camera. Speckle was distinguished from other noises due to their specific spatiotemporal characteristics that will affect correlation differently.

The phase difference dϕ between successive images is often used in interferometry to directly link the phase difference to the axial displacement $\psi_{z}$, thus cancelling the speckle noise if the noise is considered constant between successive frames. However, the presented setup uses a stroboscopic approach; therefore in Eq. (4), the speckle pattern is not stable between successive frames. In consequence, the resulting phase difference images are very noisy. Because the NCi method proposed in this paper is based on spatiotemporal cross-correlation, we are able to isolate the useful signal directly from the raw phase ϕ. Indeed, contributions from the noise have short correlation lengths compared to the signal induced by shear waves, whose wavelengths are here much larger than the speckle size. Thus, when looking at the correlation function, most of the noise can be filtered out by eliminating short correlation-length signals. The phase difference will only be used for time-of-flight processing.

### 2.3 Shear-Wave Velocity Estimation from the Displacement Field

#### 2.3.1 Classic time-of-flight algorithm

To retrieve the local speed of the waves from a propagation movie, a common approach in transient elastography is time-of-flight. By looking at the spatial displacement of the propagating wave front between different frames, the shear-wave speed can be directly measured from the spatiotemporal data or computed using correlation-based methods. The time-of-flight approach has been successfully used in most recent transient OCE papers to process wave front propagation movies.^15^^–^^26^ The major limitation of this method is the need for a known direction of shear-wave propagation and a clear wave-front, which reduces the possible mechanical sources, usually one-dimensional narrowband pulse sources, and creates artifacts in heterogeneous media caused by the change of direction of shear waves on the interfaces. Directional filtering has been successfully used to limit these artifacts.^37^ While time-of-flight is generally used with narrow temporal pulses, it has lately been extended to broadband pulses by using inverse filtering. This approach allows to significantly increase the signal-to-noise ratio (SNR),^18^ which is crucial in OCT or holography-based elastography.

In this study, a time-of-flight algorithm was implemented on simulation images. Temporal correlations were used along the direction of propagation to determine the distance traveled by the wave front between the different frames. A directional filter was used on the images prior to the time-of-flight calculations for heterogeneous media. Because of the speckle decorrelation between successive frames of the acquired digital holography movies, the quality of speed maps was deteriorated, hence a strong averaging was needed.

#### 2.3.2 Noise-Correlation-inspired approach

We propose a NCi approach to transient elastography based on previous studies in elastography using shear-wave noise-fields for ultrasound imaging^31^ and optics.^32^^–^^34^ The principle of noise-correlation elastography is that from displacement images of a diffuse shear-wave field, the field can be refocused using spatiotemporal correlation. This numerical refocusing gives quantitative access to the local stiffness. The present work uses the algorithm we developed for diffuse field elastography^34^ with spatially coherent sources, for example in the shape of pulses or chirps used in classic transient elastography. As demonstrated for seismic applications, correlation-based approaches can be used for both one-sided transient sources^38^ and two-dimensional noise-fields.^39^ The method used is explained in detail in the rest of this section.

When considering a mechanical source of shear waves s at the spatial position $\overset{\to }{r_{s}}$, the axial displacement field $\psi_{z}$ within a lossless medium at a time t and position $\overset{\to }{r}$ can be expressed using the temporal convolution ${⊗}_{t}$ as

$$\psi_{z}(\overset{\to }{r},t)=s(\overset{\to }{r_{s}},t){⊗}_{t}h(\overset{\to }{r},\overset{\to }{r_{s}},t),$$ (5)

with $h(\overset{\to }{r},\overset{\to }{r_{s}},t)$ the solution of the wave equation in the impulse regime for a receiver and a source localized, respectively, in $\overset{\to }{r}$ and $\overset{\to }{r_{s}}$. We consider the correlation C of the temporal signals from two separate points $\overset{\to }{r_{0}}$ and $\overset{\to }{r_{A}}$:

$$C(\overset{\to }{r_{0}},\overset{\to }{r_{A}},t)=\psi_{z}(\overset{\to }{r_{0}},-t){⊗}_{t}\psi_{z}(\overset{\to }{r_{A}},t).$$ (6)

By using the general expression of the axial displacement field in Eq. (5), Eq. (6) can be expressed as the auto-correlation of the source signal convoluted to the medium impulse response:

$$C(\overset{\to }{r_{0}},\overset{\to }{r_{A}},t)=s(\overset{\to }{r_{s}},-t){⊗}_{t}s(\overset{\to }{r_{s}},t){⊗}_{t}h(\overset{\to }{r},\overset{\to }{r_{s}},t){⊗}_{t}h(\overset{\to }{r_{0}},\overset{\to }{r_{s}},-t).$$ (7)

From the reciprocity principle, the cross-correlation of the signal at two points $\overset{\to }{r_{0}}$ and $\overset{\to }{r_{A}}$ is equivalent to the response at one of these points as if the second point was a source. Equation (7) can be rewritten as

$$C(\overset{\to }{r_{0}},\overset{\to }{r_{A}},t)=S(t){⊗}_{t}h(\overset{\to }{r_{0}},\overset{\to }{r_{A}},t),$$ (8)

with $S(t)=s(\overset{\to }{r_{s}},-t){⊗}_{t}s(\overset{\to }{r_{s}},t)$ the auto-correlation of the source.

In noise-correlation elastography, the source is considered infinitely broadband. In this case, the auto-correlation of the source S converges toward a Dirac distribution in Eq. (8) and the correlation function C gives direct access to the medium impulse response h. Yet, for transient elastography and in the general case, the cross-correlation of the temporal signals at different spatial positions is equivalent to the impulse response of the medium convoluted with the auto-correlation of the source. This means that in order to use temporal correlation for transient elastography, knowledge of the source temporal shape is necessary to retrieve the impulse response from the correlation C.

Under the assumptions of an isotropic lossless medium with a constant elastic-wave velocity c, the impulse function h can be expressed as a Dirac distribution δ:^40^

$$h(\overset{\to }{r_{0}},\overset{\to }{r_{A}},t)=\delta (t-\frac{\left|\overset{\to }{r_{0}}-\overset{\to }{r_{A}}\right|}{c}).$$ (9)

Under the assumptions that Eq. (9) is valid, Eq. (8) can be rewritten as

$$C(\overset{\to }{r_{0}},\overset{\to }{r_{A}},t)=S(t-\frac{\left|\overset{\to }{r_{0}}-\overset{\to }{r_{A}}\right|}{c}).$$ (10)

Equation (10) illustrates how temporal and spatial variations of the correlation function are directly linked by the mechanical wave speed c. Thus, the C function of t at $\overset{\to }{r_{0}}-\overset{\to }{r_{A}}=0$ is the same function than the C function of $\overset{\to }{r_{0}}-\overset{\to }{r_{A}}$ at t=0 at a different scale. The measure of this change of scale gives consequently quantitative access to the speed of the perturbation c, which is here the shear-wave speed $c_{\text{shear-wave}}$.

In this paper, we propose to take advantage of the simple relation between the cross-correlation C and the auto-correlation S to show that the algorithm developed for passive elastography can be applied to transient elastography. Two approaches are presented in the rest of this section.

The source auto-correlation S(t) at the position $\overset{\to }{r}=\overset{\to }{r_{0}}$ can also be expressed using the correlation function $C(\overset{\to }{r},\overset{\to }{r_{0}},t)$. The notation $C(\overset{\to }{r_{0}},\overset{\to }{r_{0}},t)$ will be used for the auto-correlation from this point.

##### Full width at half maximum method

A simple way to quantify the scale between correlation function variations in time and space is to use the full width at half maximum (FWHM). This first method consists in tracing both the cross-correlation function at t=0 and the temporal auto-correlation at $\overset{\to }{r}=\overset{\to }{r_{0}}$ and estimating the ratio of their FWHM:

$$c_{\text{shear-wave}}=\frac{\mathrm{FWHM}(C(\overset{\to }{r},\overset{\to }{r_{0}},t=0))}{\mathrm{FWHM}(C(\overset{\to }{r}=\overset{\to }{r_{0}},\overset{\to }{r_{0}},t))}.$$ (11)

In the case where the direction of propagation is unknown, the direction of the minimum FWHM is selected.

This first approach is robust; however, it requires a full spatial correlation reconstruction for each spatial position, which is time-consuming and deteriorates the spatial resolution. The FWHM method is particularly limiting with optical imaging as the size of the field of view is often too small to access full spatial correlation functions. To overcome these limitations, a second method is preferred, making use of an algorithm for passive elastography, that was developed in our previous work.^34^

##### NCi method

The method used to perform shear-wave elastography using noise-correlation is detailed in Ref. 31. This approach provides the following formula for shear-wave speed estimation:

$$c_{\text{shear-wave}}=\sqrt{\frac{V_{0}^{\mathrm{TR}}(\overset{\to }{r_{0}},\overset{\to }{r_{0}},t=0)}{\xi_{0}^{\mathrm{TR}}(\overset{\to }{r_{0}},\overset{\to }{r_{0}},t=0)}},$$ (12)

with $\xi_{0}^{\mathrm{TR}}$ the auto-correlation of the strain field and $V_{0}^{\mathrm{TR}}$ the auto-correlation of the velocity field. These quantities are also called time-reversed strain field and time-reversed particle velocity field.^31^ The time-reversed strain field and the time-reversed particle velocity field are, respectively, the spatial and temporal second derivatives of the time-reversed displacement field, which is equivalent to the correlation function :

$$\psi_{z}^{\mathrm{TR}}(\overset{\to }{r_{0}},\overset{\to }{r},t)≃\psi_{z}(\overset{\to }{r_{0}},-t){⊗}_{t}\psi_{z}(\overset{\to }{r},t)=C(\overset{\to }{r_{0}},\overset{\to }{r},t).$$ (13)

Consequently, the time-reversed fields $\xi_{0}^{\mathrm{TR}}$ and $V_{0}^{\mathrm{TR}}$ can be expressed directly in function of the correlation function:

$$\left\{ \begin{array}{c} V_{0}^{\mathrm{TR}}=\frac{\partial^{2}C}{\partial t^{2}},\\ \xi_{0}^{\mathrm{TR}}=\frac{\partial^{2}C}{\partial r^{2}}. \end{array}\right.$$ (14)

For transient elastography, the relation [Eq. (12)] can consequently be interpreted as the ratio between the temporal curvature and the spatial curvature of the correlation function at t=0 and $\overset{\to }{r}=\overset{\to }{r_{0}}$. From this ratio, the change of scale between the temporal and the spatial correlation is calculated, which corresponds to the shear-wave speed. Under the assumption that Eq. (10) can be applied, $V_{0}^{\mathrm{TR}}$ and $\xi_{0}^{\mathrm{TR}}$ can be directly linked by the shear-wave speed:

$$V_{0}^{\mathrm{TR}}=\frac{\partial^{2}C}{\partial t^{2}}=\frac{\partial^{2}C}{\partial r^{2}}.{\left(\frac{\partial r}{\partial t}\right)}^{2}=\xi_{0}^{\mathrm{TR}}.c_{\text{shear-wave}}^{2}.$$ (15)

Consequently, Eq. (13) remains true for one-dimensional signals and the algorithm developed for noise-correlation elastography^34^ can be used to process transient elastography images.

The NCi approach is based on correlations, thus is can be performed on any one-dimensional mechanical perturbation. Also, the propagation direction is intrinsically calculated by the NCi algorithm as it corresponds to the direction for which the gradient of the two-dimensional spatial correlation is minimal. The algorithm used here is the trace of the corresponding Hessian matrix. In contrast to the FWHM method that requires the full cross-correlation and auto-correlation curves, the NCi method only requires a few points of the same curves to compute the spatial derivative of the auto-correlation function at t=0. The NCi method will consequently be preferred for processing stiffness images.

### 2.4 Simulation and Sample Preparation

**Fig. 2:**
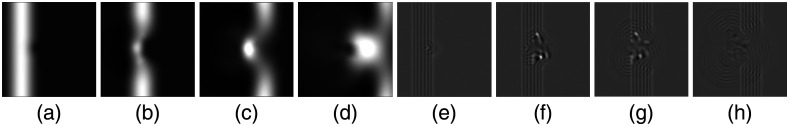
Frames from simulated propagation movies (500×500  pixels of $100\;\mu m^{2}$ with 1000 frames at 50 kHz) displayed in Video 1 and Video 2. A softer star-shaped inclusion is simulated with 6- and 3-m/s shear-wave speed for the background and the inclusion, respectively. Panels (a)–(d) correspond to frames 350, 450, 550, and 650 of Video 1, respectively, for which the shear-wave source function is a 4-ms-long half-sine. Panels (e)–(h) correspond to frames 350, 450, 550, and 650 of Video 2, respectively, where the source function is 4-ms-long noise signal (400 to 800 Hz) (Video 1, MP4, 0.20 MB [URL: https://doi.org/10.1117/1.JBO.26.8.086006.1] and Video 2, MP4, 0.29 MB [URL: https://doi.org/10.1117/1.JBO.26.8.086006.2]).

The simulated images were generated numerically using a finite difference algorithm coded in Python. 250×250  pixels propagation movies of 500 frames were created mimicking the propagation of different profiles of shear-waves sources through homogeneous media. Heterogeneous media was also simulated: 500×500  pixels and 1000-frame movies of pulse propagation through star-shaped inclusions were created to create complex structures. The frame rate of the movies is 50 kHz and the simulated pixel size is 40  μm for the homogeneous media and 100  μm for the star-shaped inclusions. Figure 2 and Video 1 and Video 2 display typical simulated propagation movies in a medium with a soft inclusion for two different shear-wave sources. Figures 2(a)–2(d) and Video 1 show the propagation of 4-ms-long half-sine shear-wave source. Figures 2(e)–2(h) and Video 2 display the propagation movie for a one-dimensional noise signal in a 400- to 800-Hz frequency band.

The tissue-mimicking samples used were $65\times 65\times 15\;{\mathrm{mm}}^{3}$ agarose gels. The mass concentration of agarose (A9539, Sigma-Aldrich, St. Louis, Missouri) in the samples ranges from 0.5% to 2% depending on the desired stiffness. Shear-wave speeds in such samples typically range from 1 to 10  m/s, which corresponds to shear-modulus between 1 and 100 kPa, similarly to biological soft tissues.^35^ Titanium dioxide (277370010, Acros Organics, Morris Plains, New Jersey) was added at a 2% mass concentration for its scattering properties. All the samples were prepared independently following the same protocol.

## 3 Results

### 3.1 Simulation

#### 3.1.1 Homogeneous media

Homogeneous media with shear-wave speeds of 2, 4, 6, 8, and 10  m/s were simulated. Different functions were tested for the mechanical stimulation: sinusoidal, hann, and sine-cube functions were generated with durations of 1, 2, and 4 ms each. The shear-wave speed was calculated from the simulated movies using the time-of-flight as well as the NCi algorithm. Each estimated shear-wave speed was calculated over the simulated area using the three different pulse durations. For all the simulations, the shear-wave speed was retrieved using the time-of-flight as well as the NCi algorithm. The speed maps retrieved were homogeneous and values were correct with negligible (<0.6%) difference between the measured and the simulated shear-wave speeds.

In the case of non-differentiable source functions such as rectangular or triangular pulses, the results from the NCi approach were not conclusive as the method is based on the calculus of derivatives. However, the spatiotemporal correlations were still calculated and the FWHM approach was successfully used to retrieve the shear-wave speed.

#### 3.1.2 Star-shaped inclusion

Star-shaped inclusions were then simulated to assess the effects of heterogeneous media on speed maps using both approaches. A shear-wave speed of 6  m/s was simulated for the background, and of 3, 4.8, 5.4, and 5.7  m/s for a soft star-shaped inclusion. Conversely, stiffer star-shaped inclusions were also generated for a 3-m/s background. A typical propagation movie is shown in Video 1 and Video 2.

**Fig. 3:**
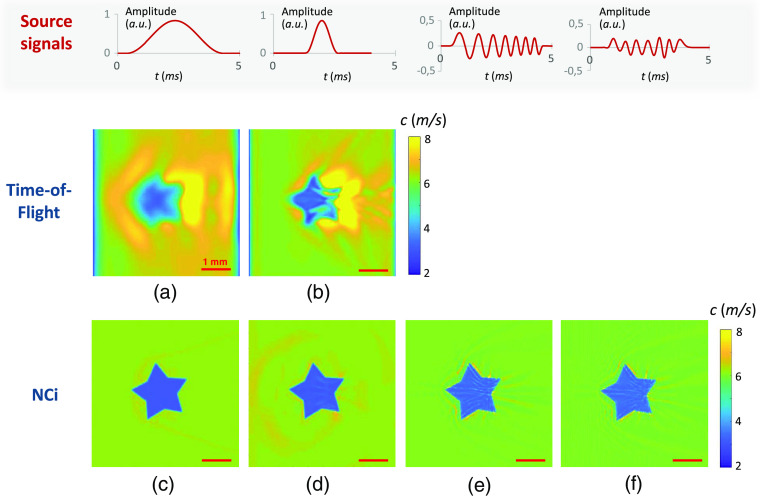
Speed maps obtained from simulated propagation movies (images of 500×500  pixels of $100\;\mu m^{2}$, 1000 frames at 50 kHz) using time-of flight [(a) and (b)] and the NCi algorithm [(c)–(f)]. A soft star-shaped inclusion was simulated with 6-m/s shear-wave speed for the background and 3  m/s for the inclusion. Different profiles of one-dimensional shear-wave sources were simulated: half-sine functions of 4 and 1.33 ms, a chirp function, and a noise signal. The profiles of the excitation sources are represented above each speed map: half sine functions [(a) and (c) for the 4-ms-long pulse; (b) and (d) for the 1.33-ms-long pulse], chirp function (e), and noise (f).

**Fig. 4:**
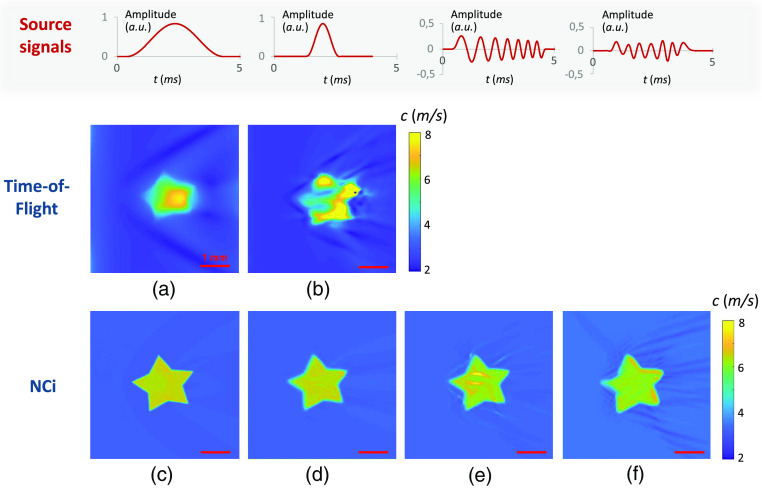
Speed maps obtained from simulated propagation movies (images of 500×500  pixels of $100\;\mu m^{2}$, 1000 frames at 50 kHz) using time-of flight [(a) and (b)] and the NCi algorithm [(c)–(f)]. A stiff star-shaped inclusion was simulated with 3-m/s shear-wave speed for the background and 6  m/s for the inclusion. Different profiles of one-dimensional shear-wave sources were simulated: half-sine functions of 4 and 1.33 ms, a chirp function, and a noise signal. The profiles of the excitation sources are represented above each speed map: half sine functions [(a) and (c) for the 4-ms-long pulse; (b) and (d) for the 1.33-ms-long pulse], chirp function (e), and noise (f).

Since the artifacts were more visible with greater stiffness contrast, shear-wave speed-maps are shown in Fig. 3 for a 3-m/s inclusion in a 6-m/s background and in Fig. 4 for the inverted contrast (6  m/s for the inclusion and 3  m/s for the background). The time-of-flight and NCi algorithms were first tested with 4- and 1.33-ms-long half-sine excitations functions [Figs. 3(a)–3(d) and 4(a)–4(d)]. To demonstrate the adaptability of the NCi algorithm to any one-dimensional excitation signal, the NCi approach was also tested with a 4-ms-long chirp excitation function from 500 to 700 Hz [Figs. 3(e) and 4(e)] and a 4-ms noise signal from 400 to 800 Hz [Figs. 3(f) and 4(f)].

The images obtained show that the inclusion can be resolved with both methods using half-sine excitation signals. Even with a directional filter,^37^ the images from the time-of-flight algorithm display specific artifacts. These artifacts stem from reflections, refraction, and scattering occurring at the interface between the background and the inclusion in both Figs. 3 and 4. On the NCi image, artifacts are largely reduced. A possible explanation is that because the predominant direction of propagation is inherently chosen locally by the NCi algorithm, changes in the propagation direction do not impact the speed values as much. The images from the NCi algorithm all display the same resolution regardless of the source signal for both stiff and soft inclusions.

The NCi algorithm was able to perform quantitative full-field stiffness measurements from heterogeneous simulation images with minor artifacts using different excitation signals. Comparison with time-of-flight images demonstrates that the NCi approach is a promising alternative to classic time-of-flight algorithms for heterogeneous media. All the obtained images also displayed super-resolution, which was expected as super-resolution has already been demonstrated for transient^28^ and passive elastography.^40^ These findings validate the NCi method and showed its potential for applications in experimental images.

### 3.2 Agarose Samples

To clarify this section, some denominations are introduced here. Two types of excitation signals were used experimentally on agarose samples. The first excitation signal consists in a repeated 1-ms-long half-sine function. The propagation of two to five pulses was recorded on each acquisition. The resulting propagation movies will be referred to as pulse acquisitions. The second type of excitation signal is a 2-ms-long chirp function, with frequencies ranging from 500 Hz to 2 kHz. The propagation of a single pulse is imaged on each acquisition. These acquisitions are referred to as chirp acquisitions in the rest of this section.

#### 3.2.1 Homogeneous media

**Fig. 5:**
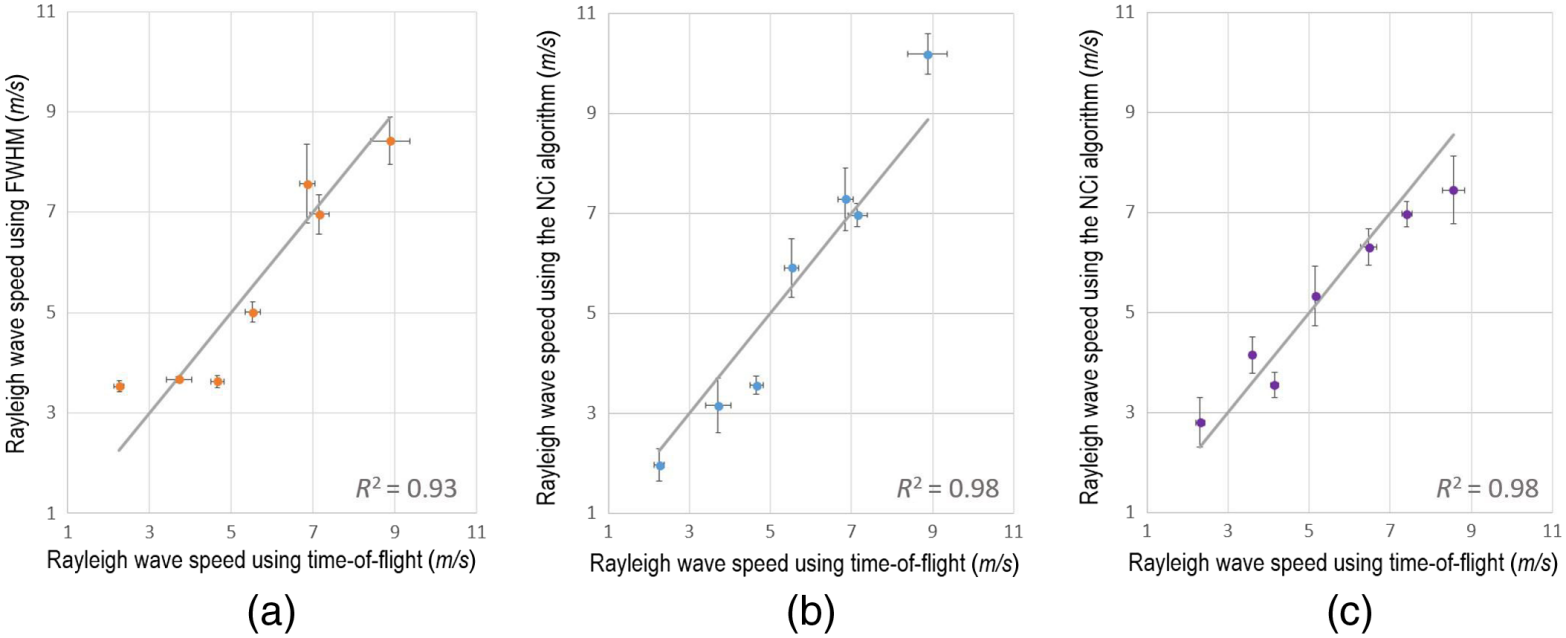
Curves representing the average speed of Rayleigh waves measured at the surface of scattering agarose samples of gradually increasing stiffness (from 0.5% to 2.0% agarose mass concentration) using different methods. Time-of-flight measures were used as a reference and presented on the abscissa axis of each graph. Figures 3(a) and 3(b) were obtained from pulse acquisitions (using four 1-ms-long half-sine excitation function). Results using the FWHM and NCi methods are presented in Figs. 3(a) and 3(b), respectively. Figure 3(c) shows the results from the NCi algorithm on chirp acquisitions (using a 2-ms-long chirp excitation function, from 500 to 2 kHz). The measures were averaged over six acquisitions for each sample. The standard deviation of the NCi and FWHM methods are presented. The gray line represents the time-of-flight measures. The correlation between time-of-flight and NCi or FWHM measures is indicated by the $R^{2}$ coefficient for each graph.

To validate both transient elastography approaches experimentally, samples with gradually varying agarose mass concentrations (0.5%, 0.75%, 1%, 1.25%, 1.5%, 1.75%, and 2%) were imaged using the digital holography setup. Pulse acquisitions and chirp acquisitions were obtained on these samples. Rayleigh wave speeds were measured consecutively with a time-of-flight method as well as with the FWHM method and the NCi algorithm. The accuracy was assessed over six acquisitions for each sample. The results are presented in Figs. 5(a) and 5(b) for the pulse acquisitions and in Fig. 5(c) for the chirp acquisitions. The Rayleigh wave speed values obtained for the samples of 0.5%, 0.75%, 1%, 1.25%, 1.5%, 1.75%, and 2% agarose mass concentration are 2.3±0.1, 3.7±0.3, 4.7±0.2, 5.5±0.2, 6.9±0.2, 7.1±0.2, and 8.9±0.5  m/s with a time-of-flight approach, respectively. Time-of-flight will be used as a reference for the FWHM and NCi approach.

While errors can be observed on the experimental data depending on the method, all the curves display a linear tendency, which means that the measurements are similar to those of time-of-flight with comparable tendencies. These results validate that the NCi approach allows for quantitative experimental measures of the phase velocity. The adaptability of the NCi algorithm to any one-dimensional excitation signal is also showcased by the experimental results from broadband and narrowband sources.

#### 3.2.2 Star-shaped inclusion

**Fig. 6:**
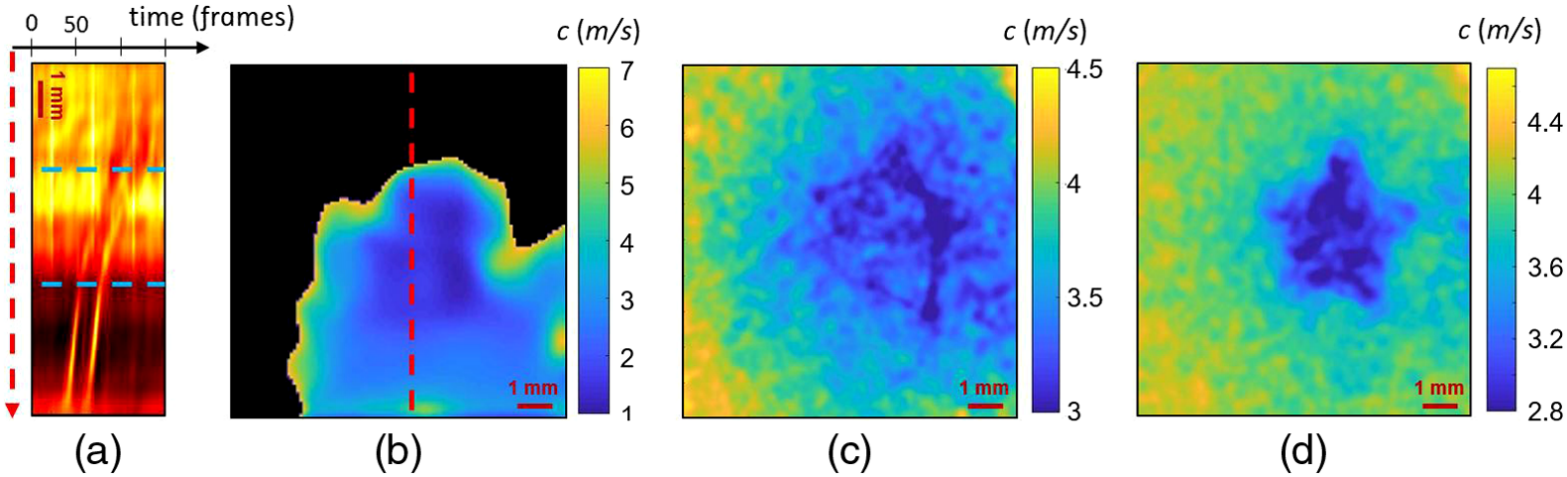
Images obtained from a 1% agarose sample with a 0.5% star-shaped agarose inclusion. Panels (a)–(c) were obtained with a pulse acquisition (using two 1-ms-long half sine source signals) and panel (d) with a chirp acquisition (using a 2-ms-long chirp source signals ranging from 500 Hz to 2 kHz). Figure 6(b) shows a speed map obtained using a time-of-flight algorithm. The black pixels represent the area where the shear-wave speed could not be calculated. Figure 6(a) represents the temporal profile of a centered vertical line, corresponding to the red line on Fig. 6(b), averaged over 20 pixels. Changes in slope are represented by blue dotted lines. Figures 6(c) and 6(d) show the speed maps retrieved from the propagation movies using the NCi algorithm.

To illustrate the feasibility of the method on inhomogeneous samples, a 1% agarose sample with a 0.5% agarose inclusion was tested. The NCi algorithm was used on the chirp and pulse acquisitions of the inclusion sample. Figure 6 shows the results of the NCi for the same star-shaped inclusion on pulse (c) and chirp (d) acquisitions. A time-of-flight speed-map could be extracted from the heterogeneous inclusions [Fig. 6(b)]. The averaged temporal profile of a centered vertical line of the propagation movie is represented in Fig. 6(a).

On the spatiotemporal representation of the pulse acquisition in Fig. 6(a), the line represents a propagating wave front. On this line, two changes of slope are visible and traced in blue: they correspond to the wave entering and exiting the inclusion. The measure of these slopes is equivalent to a time-of-flight calculation. Rayleigh wave speeds of 1.5 and 4.3  m/s were measured for the inclusion and the background using time-of-flight, respectively. The image calculated using time-of-flight [Fig. 6(b)] was strongly affected by unstable speckle noise and low signal: the inclusion is still visible but the speed could not be retrieved on the entire field-of-view. The black zone represents the area where the signal was too low to extract speed using time-of-flight. A threshold at 8  m/s was chosen to filtered out unreliable speed values. The star-shaped inclusion was resolved using the NCi method with different shapes of one-dimensional sources. An increased SNR can be noticed with the chirp acquisition [Fig. 6(d)] compared to the pulse acquisition [Fig. 6(c)]. In chirp acquisitions, the mechanical signal is indeed spread over a longer period of time than in a pulse acquisition. This consequently increases the energy of the detectable signal and the SNR.^18^ The mean Rayleigh wave speeds for the inclusions and the background are, respectively, 3.2 and 3.9  m/s for the pulse acquisition [Fig. 6(c)] and 2.9 and 4.1  m/s for the chirp acquisition [Fig. 6(d)]. The difference between the time-of-flight and the NCi speed values could stem from the low SNR of the pulse acquisition. All speed values for the inclusion and the background are coherent with the values found in Figs. 5(b) and 5(c) for homogeneous samples. Changes in the sample geometry, temperature, and humidity could explain that the measures do not correspond exactly. Speckle artifacts as well as vignetting are visible on the digital holography images.

The agarose acquisitions demonstrate the feasibility of the NCi method using digital holography images. The quantitativity was thus demonstrated on homogeneous media. It was demonstrated that inclusions can be resolved and that details smaller than the mechanical wavelength are visible. Both sinusoidal pulses and broadband mechanical stimulations were successfully tested on controlled homogeneous and heterogeneous samples.

### 3.3 Ex-Vivo Pig Liver

**Fig. 7:**
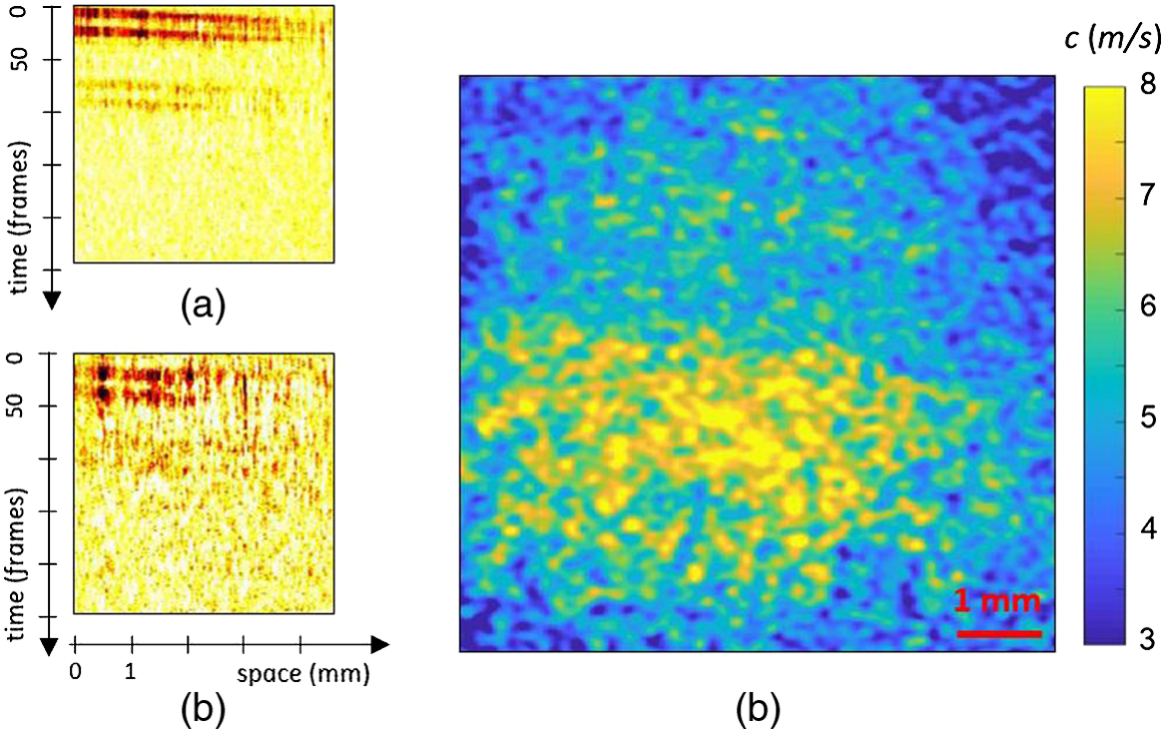
The spatiotemporal representations of a 1-ms-long pulse propagating in a cooked and raw pork liver sample are presented in (a) and (b), respectively. The speed map retrieved from the same set of propagation images using the NCi approach is shown in (c).

As the objective of this work is biological tissue imaging, a pig liver sample was tested with the NCi method. Half of the sample was cooked to alter its mechanical properties and the other half was left raw. Similarly, to the agarose samples, 1-ms-long half-sine function was used. Figures 7(a) and 7(b) display the spatiotemporal images of the wave fronts obtained for the propagation of the 1-ms-long impulses.

In the raw and cooked liver, the Rayleigh wave speeds were 3.6 and 6.3  m/s using time-of-flight, respectively. The equivalent wavelength map was computed using the NCi approach, as shown in Fig. 7(c). The obtained speed map shows contrast between both parts of the samples with mean equivalent Rayleigh wave speeds of 3.5  m/s and 4.5  m/s. The Rayleigh wave speeds obtained with the NCi method on raw and cooked liver correspond, respectively, to shear modulus of 14 and 22 kPa. As a rough comparison, previous work on the human liver presented elasticity measures between 1 and 50 kPa with ultrasounds and mechanical methods.^41^

## 4 Conclusion

In this study, quantitative full-field transient elastography was performed successfully using a digital holography setup with two transient elastography approaches: time-of-flight and NCi. To our knowledge, this is the first time that transient elastography is coupled with a digital holography setup. The time-of-flight and NCi methods were tested on simulation images and experimentally validated on agarose samples with controlled optical and mechanical properties. These tests yielded quantitative results as well as coherence between the different approaches. The first results on biological tissue are also presented.

The NCi method was introduced to perform transient elastography using spatiotemporal correlations. This new NCi method has the advantage of being simple and adapted to any derivable one-dimensional excitation signal and is fully compatible with non-contact mechanical sources, such as air-puffs ^22^^,^^23^^,^^25^ or focused ultrasounds.^19^^–^^21^ The NCi algorithm is particularly interesting for full-field images as, contrarily to time-of-flight, it does not require any additional propagation direction calculation or propagative filter. The propagation direction is indeed intrinsically taken into account and reflections do not negatively impact the speed-map processing. The NCi method, like the time-of-flight, yielded quantitative results and achieved super-resolution.

The NCi method is based on derivatives, although taken after correlation, derivative calculations tend to decrease the SNR, which could be improved by taking advantage of the correlation length of the different noises. Moreover, the stroboscopic approach presented in this paper introduced an unstable speckle noise on the acquisitions, which made speed map processing particularly hard with time-of-flight. Using a high-frame rate camera would allow better results with both time-of-flight and NCi methods and the speed maps could be imaged in real-time. Because of the cost of high frame-rate cameras and the lower SNR, a stroboscopic approach was preferred in this study.

On the processed speed maps, the time-of-flight approach presented artifacts, as well as the NCi method to a lesser extent. These artifacts are localized near stiffness discontinuities, where reflections, refraction, and scattering are particularly visible on the propagation movies. The simulations notably emphasized the criticality of discontinuous media for shear-wave transient elastography. The relation between the measured shear-wave speed and the stiffness of the medium presented in Eq. (3) should be used cautiously for heterogeneous media.

Contrarily to time of flight, the NCi method could also be used without the high temporal sampling frequency. The temporal dimension of the propagation movies is used here by the NCi algorithm to compute the temporal curvature of the source auto-correlation although the pulse sent to the actuator is programmed, so its temporal shape is known. In this paper, we used the spatiotemporal data from the propagation movie because the coupling between the piezoelectric actuator and the agarose sample was not consistent enough to use the signal transmitted to the actuator. However, if a reliable coupling between the actuator and the sample is achieved, a NCi algorithm could retrieve quantitative speed maps from a stack of wave front images acquired with a low frame-rate camera and without synchronization. In this paper, we described an adaptation of the noise-correlation approach developed for diffuse-field elastography to spatially coherent sources used for classical transient elastography. Passive elastography is a very promising method for full-field imaging systems as it achieves quantitative two-dimensional stiffness mapping with a low frame-rate. However, passive elastography is not optimal for scanning imaging systems such as OCT, which are used in a number of clinical applications: classical transient elastography is thus usually preferred. We showed that the formalism used for processing noise-correlation elastography could be adapted to spatially coherent sources, bringing a number of advantages and bridging the gap between passive and classical transient elastography approaches.
